# The Cross Talk Between p53 and mTOR Pathways in Response to Physiological and Genotoxic Stresses

**DOI:** 10.3389/fcell.2021.775507

**Published:** 2021-11-18

**Authors:** Danrui Cui, Ruirui Qu, Dian Liu, Xiufang Xiong, Tingbo Liang, Yongchao Zhao

**Affiliations:** ^1^ Department of Hepatobiliary and Pancreatic Surgery, The First Affiliated Hospital, Zhejiang University School of Medicine, Hangzhou, China; ^2^ Zhejiang Provincial Key Laboratory of Pancreatic Disease, The First Affiliated Hospital, Zhejiang University School of Medicine, Hangzhou, China; ^3^ Cancer Center, Zhejiang University, Hangzhou, China; ^4^ Institute of Translational Medicine, Zhejiang University School of Medicine, Hangzhou, China; ^5^ Cancer Institute of the Second Affiliated Hospital, Zhejiang University School of Medicine, Hangzhou, China

**Keywords:** p53, mTOR, transcription, miRNA, tumorigenesis, MDM2, post-translation

## Abstract

The tumor suppressor p53 is activated upon multiple cellular stresses, including DNA damage, oncogene activation, ribosomal stress, and hypoxia, to induce cell cycle arrest, apoptosis, and senescence. Mammalian target of rapamycin (mTOR), an evolutionarily conserved serine/threonine protein kinase, serves as a central regulator of cell growth, proliferation, and survival by coordinating nutrients, energy, growth factors, and oxygen levels. p53 dysfunction and mTOR pathway hyperactivation are hallmarks of human cancer. The balance between response to stresses or commitment to cell proliferation and survival is governed by various regulatory loops between the p53 and mTOR pathways. In this review, we first briefly introduce the tumor suppressor p53 and then describe the upstream regulators and downstream effectors of the mTOR pathway. Next, we discuss the role of p53 in regulating the mTOR pathway through its transcriptional and non-transcriptional effects. We further describe the complicated role of the mTOR pathway in modulating p53 activity. Finally, we discuss the current knowledge and future perspectives on the coordinated regulation of the p53 and mTOR pathways.

## Introduction: The Tumor Suppressor p53

p53, a well-known tumor suppressor, acts as a “guardian of the genome” to maintain genome stability and cellular homeostasis (Vousden and Prives, 2009; Hafner et al., 2019). Upon induction of various cellular stresses, especially DNA damage, p53 is activated to induce cell cycle arrest, apoptosis, and senescence which suppress tumorigenesis by eliminating damaged and potentially precancerous cells. p53 is the most frequently mutated gene in human cancers (Hainaut and Hollstein, 2000). Approximately 50% of human tumors carry p53 mutations, and in over 80% of the tumors the p53 pathway is dysfunctional (Ozaki and Nakagawara, 2011). As a transcription factor, p53 exerts tumor-suppressive effects by directly binding to specific DNA sequences to activate or repress the transcription of target genes, such as *MDM2* (Barak et al., 1993), *p21* (el-Deiry et al., 1993), *NOXA* (Oda et al., 2000), *PUMA* (Nakano and Vousden, 2001), and *RPS27L* (He and Sun, 2007; Li et al., 2007) [for review, see (Fischer, 2017)]. The canonical p53 binding sequence RRRC (A/T) (A/T)GYYY(N)_0-13_RRRC (A/T) (A/T)GYYY (R: A or G, Y: C or T, and N: any nucleotide) is normally located near the transcription start site (Hafner et al., 2019).

Given the key role of p53 in tumorigenesis, the protein levels and activity of p53 are precisely regulated by multiple regulators [for review, see (Kruse and Gu, 2009; Chao, 2015)], among which the E3 ubiquitin ligase, mouse double minute 2 homolog (MDM2), is the pivotal negative regulator (Hock and Vousden, 2014). Under non-stressed conditions, MDM2 binds specifically to p53 and promotes the ubiquitination and proteasomal degradation of p53, which maintains p53 at low levels. In response to stress signals, such as DNA damage, certain kinases, such as ATM and CHK2, are activated to phosphorylate p53 and abolish the interaction between p53 and MDM2, leading to p53 stabilization and transcriptional activation or repression of downstream target genes. When DNA repair is completed, p53 returns to basal levels, and subsequently, the cell cycle is restored to normal progression. The E3 ligase F-box and WD repeat domain-containing 7 (FBXW7) plays an important role in p53 turnover during DNA damage recovery (Galindo-Moreno et al., 2019; Tripathi et al., 2019; Cui et al., 2020b).

p53 dysfunction and hyperactivation of the mTOR pathway are hallmarks of human cancer (Hanahan and Weinberg, 2011). Accumulating evidence indicates that the tumor suppressor p53 regulates the machinery of the mTOR pathway at multiple levels to control a broad array of cellular processes, including cell proliferation, apoptosis, autophagy, migration, and tumorigenesis. In this review, we summarize the current knowledge regarding the role of p53 in the regulation of the mTOR pathway through transcription-dependent and transcription-independent mechanisms. A thorough understanding of the interplay between p53 and the mTOR pathway will shed light on the development of novel strategies for cancer therapy.

### The mTOR Pathway

**FIGURE 1:**
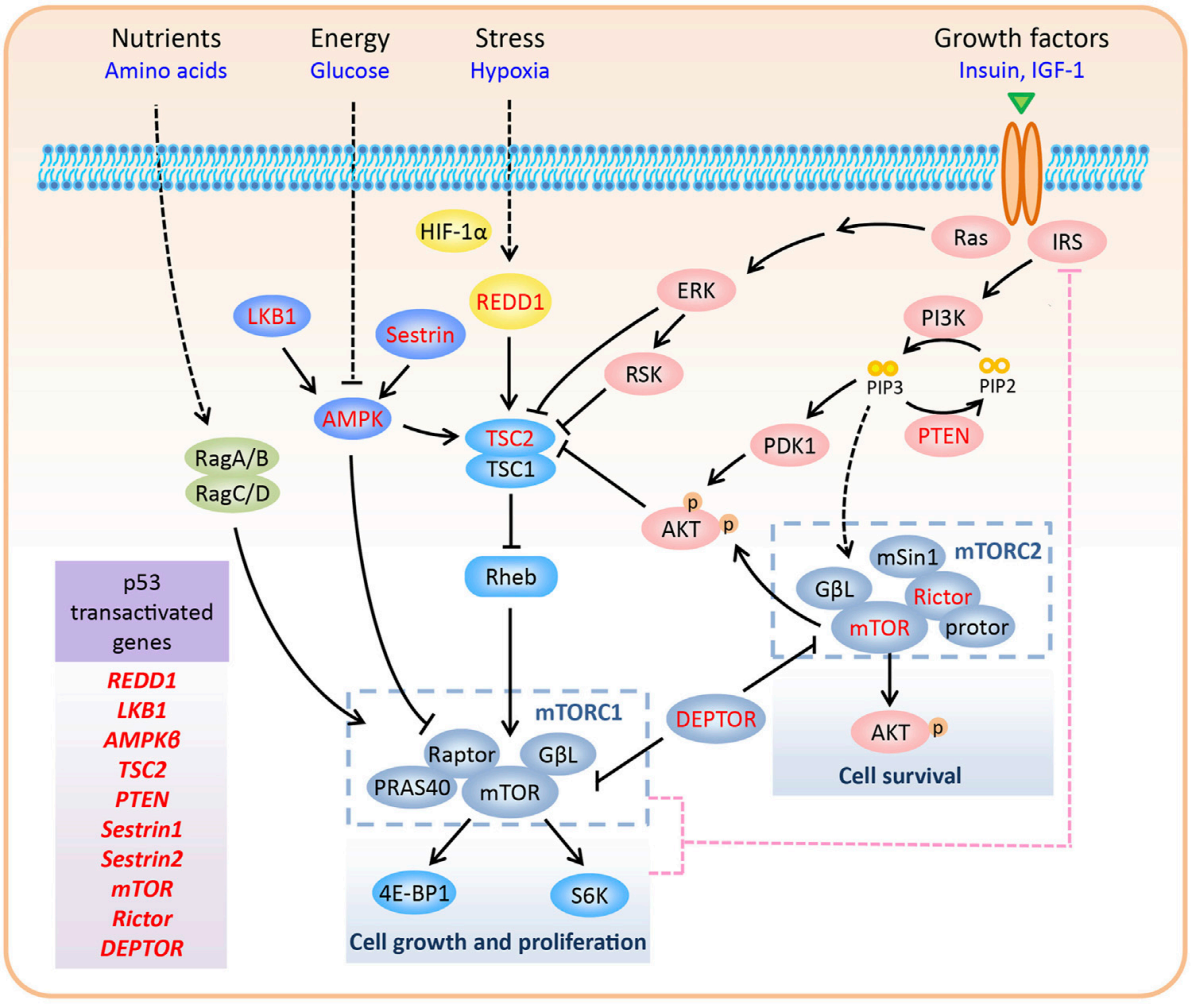
Regulation of the mTOR pathway. In mammalian cells, mTOR forms two complexes with distinctive structure and functions, namely mTORC1 and mTORC2. mTORC1 promotes cell growth and proliferation via phosphorylation of S6K and 4E-BP1. In contrast, mTORC2 regulates cell survival by phosphorylating Ser473 of AKT. mTORC1 responds to various signals, including nutrients, energy, growth factors, and oxygen levels. Upon negative feedback from mTORC1/S6K to IRS1/PI3K signaling, hyper-activated mTORC1 inhibits the activity of mTORC2. Various components of the mTOR pathway are directly transactivated by p53, including REDD1, LKB1, AMPKβ, TSC2, PTEN, Sestrin1/2, mTOR, Rictor, and DEPTOR (highlighted in red color in the figure).

The mammalian target of rapamycin (mTOR), an evolutionarily conserved serine/threonine protein kinase, is a member of the phosphoinositide-3-kinase (PI3K)-related kinase (PIKK) family, along with ATM, ATR, DNA-PK, and SMG-1 (Lovejoy and Cortez, 2009). While all members of the family are involved in DNA damage response, mTOR also responds to various other signals, including nutrients, energy, growth factors, and oxygen levels. By integrating both extracellular and intracellular signals, mTOR coordinates cellular anabolic and catabolic processes, including cell growth, proliferation, survival, and autophagy (Figure 1).

#### mTOR and its Effectors

In mammalian cells, mTOR forms two complexes with distinctive structures and functions, namely mTORC1 and mTORC2. mTORC1 is composed of mTOR, Raptor, PRAS40, and GβL, whereas mTORC2 consists of mTOR, Rictor, mSin1, protor, and GβL (Zhao and Sun, 2012; Saxton and Sabatini, 2017). Although the mTOR protein, which is present in both the complexes, was discovered as an interacting protein of the rapamycin-FKBP12 complex, only mTORC1 is sensitive to rapamycin inhibition (Sabatini et al., 1994). Recently, the core structure of mTORC2 has been resolved, and it has been shown that the rapamycin-FKBP12 binding domain in mTOR is masked by the C-terminus of Rictor, thus resolving the rapamycin insensitivity of mTORC2 (Scaiola et al., 2020). DEP domain-containing mTOR-interacting protein (DEPTOR) inhibits mTORC1 and mTORC2 by directly binding to mTOR via its PDZ domain (Peterson et al., 2009). mTORC1 has two major substrates, S6K and 4E-BP1, which regulate several aspects of mRNA translation (Sengupta et al., 2010). Thus, mTORC1 promotes cell growth and proliferation by modulating phosphorylation-dependent mRNA translation. However, the effectors of mTORC2 are primarily AGC kinases, including AKT, PKC, and SGK1 (Fu and Hall, 2020). mTORC2 regulates cell survival by phosphorylating Ser473 of AKT, the best-characterized mTORC2 substrate. Compared to mTORC1, much is still unknown about the upstream regulators of mTORC2 (Figure 1). Thus, in the next section, we focus on the regulation of mTORC1 in response to various signals.

#### Regulation of mTORC1 by Upstream Signals

Upstream signals regulate mTORC1 activity mainly through two mechanisms: by direct control of mTORC1 components or by the supervision of Rheb GTPase, which interacts with and activates mTORC1 (Long et al., 2005). Tuberous sclerosis complex 2 (TSC2), a GTPase-activating protein (GAP) for Rheb, together with its partner TSC1, inactivates Rheb GTPase (Inoki et al., 2003). Below, we describe the manner in which mTORC1 is either activated or inactivated by distinct upstream signals.

1) Growth factors: Growth factors, such as insulin and insulin-like growth factor 1 (IGF-1), promote cellular anabolic processes by activating mTORC1. The binding of insulin/IGF-1 to its receptor recruits insulin substrate 1 (IRS1) to the receptor and activates PI3K, which phosphorylates PIP2 to PIP3. PIP3 recruits AKT to the plasma membrane, where it is fully activated by direct phosphorylation of Thr308 by PDK1 and Ser473 by mTORC2. During growth factor signaling, AKT (Inoki et al., 2002; Manning et al., 2002; Potter et al., 2002), along with other kinases such as ERK and RSK (Roux et al., 2004; Ma et al., 2005), phosphorylates and inhibits TSC2, leading to the activation of Rheb and mTORC1. Growth factor-activated AKT also stimulates mTORC1, independent of TSC2. AKT can directly phosphorylate proline-rich AKT substrate of 40 kDa (PRAS40), a negative regulator of mTORC1, that inhibits the interaction between mTORC1 and its substrates, and dissociates itself from mTORC1 (Sancak et al., 2007; Haar et al., 2007).

2) Glucose and energy: Glucose deprivation decreases glycolytic flux and inhibits mTORC1 by lowering ATP levels. LKB1 and AMPK are two major upstream kinases of the mTOR pathway and are involved in monitoring the levels of glucose and energy (ATP and AMP) (Inoki et al., 2003; Shaw et al., 2004; Gwinn et al., 2008). In response to increased AMP/ATP ratio, LKB1 phosphorylates AMPKα (the catalytic subunit of AMPK) on Thr172 to activate AMPK. Activated AMPK phosphorylates TSC2 (Inoki et al., 2003) and Raptor (Gwinn et al., 2008) to inhibit mTORC1.

3) Hypoxia: Under hypoxic conditions, HIF-1α promotes the expression of REDD1 to inhibit mTORC1 by activating the TSC1-TSC2 complex (Brugarolas et al., 2004). Mechanistically, REDD1 may release TSC2 from its inhibitory protein 14-3-3, thus facilitating the interaction between TSC1 and TSC2 (DeYoung et al., 2008; Vega-Rubin-de-Celis et al., 2010). In addition to hypoxia, REDD1 is induced by several cellular stressors, including reactive oxygen species (ROS), glucocorticoids, DNA damage, and heat shock (Ellisen et al., 2002; Wang et al., 2003), indicating a universal function of REDD1 in coordinating stress signals to mTORC1.

4) Amino acid: Amino acid signaling recruits and activates mTORC1 to the lysosomal membrane, where a pool of Rheb resides. In this process, the Rag GTPase heterodimer (consisting of RagA/B and RagC/D), which is activated by amino acids, serves as a scaffold for mTORC1 via interacting with Raptor (Sancak et al., 2008; Sancak et al., 2010).

#### Feedback Loop Between mTORC1 and mTORC2

As a negative regulator of IRS1, mTORC1 also acts upstream of the PI3K-AKT pathway to inhibit mTORC2. This negative feedback loop is initiated through multiple mechanisms. First, S6K, a substrate of mTORC1, decreases the activity and protein levels of IRS1 by phosphorylating it (Harrington et al., 2004; Um et al., 2004; Shah et al., 2004). Second, mTORC1 phosphorylates and stabilizes growth factor receptor-bound protein 10 (Grb10), which inhibits IRS1/2 phosphorylation and destabilizes IRS1 (Hsu et al., 2011; Yu et al., 2011). Third, mTORC1 directly phosphorylates IRS1 at sites that inhibit its interaction with PI3K (Tzatsos, 2009). Therefore, high levels of DEPTOR, a natural inhibitor of both mTORC1 and mTORC2, inhibit mTORC1 and activate mTORC2 by relieving the feedback inhibition from mTORC1 to IRS1/PI3K signaling (Peterson et al., 2009; Zhao et al., 2011; Zhao and Sun, 2012; Cui et al., 2020a) (Figure 1).

### Regulation of the mTOR Pathway by p53

To maintain normal cell growth and proliferation, it is important for cells to coordinate stimulatory signals (such as nutrients, energy, and growth factors) and inhibitory stresses (such as DNA damage and hypoxia). The tumor suppressor p53, a stress-induced transcription factor, can inhibit cell growth and proliferation via its target genes, such as the cyclin-dependent kinase (CDK) inhibitor p21, which serves as a cell cycle inhibitor (Levine, 1997). More recently, elucidating the mechanism of p53 in directly regulating the mTOR pathway has become an attractive area of research due to the critical roles of p53 and mTOR in tumorigenesis. In the following sections, we discuss the emerging roles of p53 in controlling the mTOR pathway through its transcriptional and non-transcriptional effects.

#### Target Genes of p53 in the mTOR Pathway

Upon multiple stresses, p53 is activated to inhibit cell growth and proliferation, which undergo high error rates upon induction of stress. Thus, inhibition of mTORC1, which promotes cell growth and proliferation, is an important hallmark of the cellular stress response. Actually, multiple negative regulators of mTORC1, as discussed earlier, are direct transcriptional targets of p53, including *REDD1* (Ellisen et al., 2002), *LKB1* (Co et al., 2014; Xie et al., 2017), *AMPKβ* (a regulatory subunit of AMPK) (Feng et al., 2007), and *TSC2* (Feng et al., 2007). Moreover, *PTEN*, which encodes a phosphatase that catalyzes PIP3 to PIP2 to inactivate the PI3K-AKT pathway, contains p53 binding sites and is transactivated by p53 (Stambolic et al., 2001). Finally, upon genotoxic stress, p53 also promotes the transcription of Sestrin1 and Sestrin2 to inhibit mTORC1, through activation of AMPK and TSC2 (Budanov and Karin, 2008) (Figure 1 and Table 1).

**TABLE 1:** Target genes of p53 involved in mTOR signaling.

Targets	Position	Sequence	Refs
REDD1	–601 ∼ –582	AAA​CAA​GTC​TTT​CCT​TGA​TC	Ellisen et al. (2002)
LKB1	–108 ∼ –88	AAC​CAA​CGG​GTG​GGC​ACG​TCG	Co et al. (2014); Xie et al. (2017)
AMPKβ	Exon 1	GTT​CTT​GCC​GCG​GCT​TGC​CT	Feng et al. (2007)
TSC2	Intron 2a	AGGCTAGTCTGAAACTCCTGGGCTGACGTGACGGGCATGGTGGCACATGCCT	Feng et al. (2007)
	Intron 2b		
	Intron 11	TAACAAGCTCGGGGCTAGCCC	
PTEN	–1190 ∼ –1157	GAGCAAGCCCCAGGCAGCTACACTGGGCATGCTC	Stambolic et al. (2001)
Sestrin1 (PA26)	–1241 ∼ –1222	GGA​CAA​GTC​TCC​ACA​AGT​CA	Velasco-Miguel et al. (1999)
Sestrin2 (Hi95)	Not identified	Not identified	Budanov et al. (2002)
mTOR	0.5 kb upstream promoter	Not identified	Ge et al. (2019)
Rictor	0.5 kb upstream promoter	Not identified	Ge et al. (2019)
DEPTOR	–196 ∼ –169	GCTCAAGTTTCTGGGGCCGGACTAGCCC	Cui et al. (2020a)

Contrary to the inhibitory effect of p53 target genes in controlling mTORC1, the function of these genes in mTORC2 seems much more complex, being highly cell- and context-dependent. In the alternative lengthening of telomeres (ALT) cancer cells (such as U2OS cells), p53 stimulates the transcription of mTOR and Rictor, two important components of mTORC2, to activate AKT and inhibit apoptosis (Ge et al., 2019). Recently, our group reported that DEPTOR is a direct target of p53 and its expression is positively correlated with p53 activity, both in cultured cancer cells and mouse tissues under normal conditions, and is further induced by activated p53 under genotoxic conditions. Given that DEPTOR inhibits both mTORC1 and mTORC2, and there is a negative feedback from mTORC1 to IRS1/PI3K signaling, p53-mediated DEPTOR expression has distinct roles in regulating mTORC2 under non-stressed and genotoxic stress conditions. In non-stressed cells, p53-mediated DEPTOR expression inhibits mTORC2 activity, which is reflected by the decreased phosphorylation of AKT at Ser473; whereas, upon genotoxic treatment, the dramatic induction of DEPTOR expression via p53 hyperactivation inhibits mTORC1, subsequently alleviating the feedback inhibition from mTORC1 to IRS1, thereby activating mTORC2 via IRS1/PI3K signaling (Cui et al., 2020a) (Figure 1 and Table 1).

#### p53 Regulates the mTOR Pathway via microRNAs

The discovery of microRNAs (miRNA or miR) has added another layer of complexity to the regulation of the mTOR pathway by p53. miRNAs are a class of endogenously expressed small non-coding RNAs (17–24 nucleotides) that regulate the expression of multiple genes at the post-transcriptional level (Macfarlane and Murphy, 2010). miRNAs inhibit protein expression by enhancing mRNA degradation or suppressing translation via partial base pairing with the 3′-untranslated region (3′-UTR) of the target mRNA of protein coding genes (Lewis et al., 2005). miRNAs play critical roles in several biological processes, including proliferation, survival, metastasis, and stemness. In particular, overexpression of oncogenic miRNAs or downregulation of tumor-suppressive miRNAs contributes to tumorigenesis (Babashah and Soleimani, 2011). It is well established that p53 is an important regulator of miRNAs (Hermeking, 2007). Global sequence analysis showed that more than 46% of the 326 miRNA promoters contain putative p53 binding sites in HCT116 cells (Xi et al., 2006). In addition, various miRNAs have been identified as direct transcriptional targets of p53 and many of them are involved in p53-mediated tumor-suppressive functions (Hermeking, 2012). Moreover, besides the regulation of miRNAs at the transcriptional level, p53 promotes the maturation of certain miRNAs at the post-transcriptional level (Suzuki et al., 2009). Conversely, many miRNAs directly downregulate p53 protein levels by binding to the 3′-UTR of p53 mRNA (Liu et al., 2017).

Accumulating evidence shows that a large number of miRNAs act opposingly on the mTOR pathway, which is often hyperactivated in cancers (Zhang et al., 2017). Thus, p53 exerts its control on the mTOR pathway via miRNAs. Among all miRNAs, members of the miRNA-34 family (miR-34a/b/c) have been identified as the most common targets of p53 with the highest induction by activated p53 (Hermeking, 2007). Overexpression of miR-34a in prostate cancer cells inhibited the phosphorylation of AMPK and upregulated the phosphorylation of mTOR. As a result, miR-34a sensitizes cancer cells to chemotherapy by inhibiting autophagy through the AMPK-mTOR axis (Liao et al., 2016). However, the direct target(s) of miR-34 in regulating the mTOR pathway remains to be elucidated. Currently, some other miRNAs, directly regulated by p53, are emerging as vital regulators of the mTOR pathway at the post-transcriptional level, and these miRNAs, downstream of p53, are as follows (Table 2):

**TABLE 2:** p53 regulates the mTOR pathway via microRNAs.

miRNAs	p53-mediated regulation of miRNA	The effects of p53 on miRNA expression	miRNA targets in mTOR signaling	The effects on mTOR signaling	Refs
miR-34	Transcriptional	Upregulation	Unknown	Activation	Hermeking. (2007); Liao et al. (2016)
miR-100	Transcriptional	Downregulation	mTOR	Suppression	Sun et al. (2013); Xu et al. (2013); Zhang et al. (2014a); Ghose and Bhattacharyya (2015)
miR-101	Post-transcriptional	Upregulation	mTOR	Suppression	Lin et al. (2014); Fujiwara et al. (2018)
miR-145	Transcriptional	Upregulation	S6K1	Suppression	Sachdeva et al. (2009); Xu et al. (2012)
miR-149	Transcriptional	Upregulation	AKT1	Suppression	Jin et al. (2011); Zhang et al. (2014b)
miR-155	Transcriptional	Upregulation	Rheb, Rictor, and S6K2	Suppression	Wang et al. (2013); Wan et al. (2014); Wang et al. (2018)
miR-199a-3p	Post-transcriptional	Upregulation	mTOR	Suppression	Fornari et al. (2010); Wang et al. (2012a); Wu et al. (2013)

1) miR-100: miR100, a member of the miR-99 family (including miR-99a, miR-99b, and miR-100), is negatively regulated by p53. p53 binds to the upstream sequences of miR-100 and suppresses its transcription in both mouse striatal cells and human cervical carcinoma HeLa cells (Ghose and Bhattacharyya, 2015). miR-100 inhibits the expression of mTOR by directly targeting its 3′-UTR and acts as a tumor suppressor in esophageal squamous cell carcinoma (ESCC) (Sun et al., 2013; Zhang N. et al., 2014) and bladder cancer (Xu et al., 2013). Thus, p53 may activate the mTOR pathway by inhibiting the transcription of miR-100 in certain types of cancer.

2) miR-101: miR-101 is downregulated in various cancers, including ovarian cancer, prostate cancer, hepatocellular carcinoma, bladder transitional cell carcinoma, gastric cancer, and non-small cell lung cancer, and is negatively associated with the progression and invasion of malignancies, such as prostate cancer (Gui and Shen, 2012). In human osteosarcoma cells, miR-101 directly targets mTOR and decreases its expression, resulting in the suppression of cell proliferation and induction of apoptosis (Lin et al., 2014). Interestingly, p53 promotes the maturation of miR-101 at the post-transcriptional level (Fujiwara et al., 2018). Therefore, p53 may inhibit the mTOR pathway by post-transcriptional activation of miR-101.

3) miR-145: miR-145, a direct target of p53, binds to the 3′-UTR of c-MYC and inhibits its expression, thereby repressing cancer cell growth both *in vitro* and *in vivo* (Sachdeva et al., 2009). Moreover, miR-145 suppresses S6K1 expression at the post-transcriptional level to inhibit tumorigenesis and tumor angiogenesis (Xu et al., 2012). Furthermore, p53-mediated transcription of miR-145 may suppress tumor growth by cooperatively inhibiting the oncogenic functions of c-MYC and the mTOR pathway.

4) miR-149: miR-149 plays a dual role, that is controversial, either as a tumor suppressor or as an oncogene in different types of cancer (Wang Y. et al., 2012). miR-149 inhibits the tumorigenesis of hepatocellular carcinoma (HCC) via directly targeting AKT1 to regulate the AKT/mTOR pathway (Zhang Y. et al., 2014). However, miR-149, which is directly upregulated by p53, acts as an oncogenic regulator in melanoma cells by targeting glycogen synthase kinase 3α (GSK3α) to stabilize MCL-1 and inhibit apoptosis (Jin et al., 2011). Thus, it will be intriguing to characterize the unique role of the p53-miR-149-AKT/mTOR axis in different types of tumors.

5) miR-155: miR-155 targets several components of the mTOR pathway, including Rheb, Rictor, and S6K2, by directly binding to their 3′-UTRs (Wang et al., 2013; Wan et al., 2014). By interfering with both mTORC1 and mTORC2 signals, miR-155 suppresses cell proliferation, activates autophagy, and induces G1/S cell cycle arrest. Additionally, it has been reported that under high glucose conditions, p53 directly promotes miR-155 expression as a transcription factor in human renal proximal tubule (HK-2) cells (Wang et al., 2018). However, the role of p53 in regulating the transcription of miR-155 under normal conditions or upon glucose deprivation, and the functions of the p53-miR-155-mTOR pathway in physiological and pathological processes remain largely unknown.

6) miR-199a-3p: miR-199a-3p is upregulated by p53 at the post-transcriptional level (Wang J. et al., 2012). miR-199a-3p directly interacts with the 3′-UTR of mTOR and inhibits the mTOR pathway and restrains endometrial cancer cell proliferation (Wu et al., 2013) as well as increases the sensitivity of HCC cells to doxorubicin-induced apoptosis (Fornari et al., 2010). Given that p53 is highly activated in response to DNA damage, due to genotoxic treatments (e.g., doxorubicin), it is probable that the p53-miR-199a-3p-mTOR pathway regulates cancer cell survival during chemotherapy or radiotherapy.

#### Non-Transcriptional Effects of p53 in Regulating the mTOR Pathway

In general, the tumor suppressor p53 regulates various cellular processes via *trans*-activating or *trans*-repressing downstream gene expression as a transcription factor. Interestingly, in recent decades, several studies have shown that in addition to its activity in the nucleus, p53 exhibits transcription independent functions in the cytoplasm (Comel et al., 2014). The best-characterized extranuclear function of p53 is the induction of apoptosis. It has been reported that overexpression of a truncated murine p53 (p53dl214), containing only 214 amino acid residues of the N-terminus and lacking DNA-binding activity, could trigger extensive apoptosis in HeLa cells as well (Haupt et al., 1995). Mechanistically, upon apoptotic induction, p53 translocates from the nucleus to the mitochondrial outer membrane, and interacts with pro-survival Bcl-2 family members (such as Bcl-w and Bcl-X_L_) to release pro-apoptotic Bcl-2 proteins (such as Bax and Bak) to induce apoptosis (Vaseva and Moll, 2009; Czabotar et al., 2014). Moreover, cytosolic p53 regulates autophagy via the mTOR pathway.

Autophagy, a cellular catabolic process that recycles unwanted proteins and damaged organelles in the lysosomes, is regulated by two biologically significant molecules: mTOR and AMPK. mTORC1 inhibits autophagosome formation by phosphorylating ULK1 at Ser757 to suppress the ULK1 complex (Kim et al., 2011), and mTORC2 restrains the transcription of several *ATG*s via AKT-FoxO3 signaling to inhibit autophagy (Guertin et al., 2006; Zhao et al., 2008). However, AMPK plays a positive role in autophagy induction. On one hand, AMPK can phosphorylate TSC2 and Raptor to inhibit mTOR (Inoki et al., 2003; Gwinn et al., 2008); on the other hand, AMPK can directly phosphorylate ULK1 at Ser317 and Ser777 to activate the ULK1 complex and initiate autophagy (Kim et al., 2011). Notably, the tumor suppressor p53 has a dual role in the regulation of autophagy. As a transcription factor, p53 transactivates several genes that induce autophagy, including *TSC2* (Feng et al., 2007), *AMPKβ1* (Feng et al., 2007), *Sestrin1/2* (Budanov and Karin, 2008), and *DRAM* (Crighton et al., 2006). However, cytosolic p53, either the wild-type or mutant form, represses autophagy (Tasdemir et al., 2008a; Tasdemir et al., 2008b). In fact, various known autophagy-inducing stimuli, such as rapamycin treatment or ER stress, cause the cytoplasmic translocation of p53, which is subsequently degraded via MDM2-mediated ubiquitination (Pluquet et al., 2005; Yorimitsu et al., 2006). Consistently, pharmacological inhibition or depletion of p53 induces autophagy in nematodes, mice, and human cells under normal conditions (Tasdemir et al., 2008b). Moreover, p53 suppresses autophagy through a non-transcriptional effect via cytoplasmic localization, which is supported by the following evidence: 1) expression of both wild-type and ER-targeted p53 inhibited high levels of basal autophagy in HCT116 *p53* ^−/−^ cells; 2) expression of nuclear p53 (disturbed NES by L348A and L350A) failed to inhibit autophagy; and 3) a point mutation (R175H) in p53 that induces a conformational change, abrogated the autophagy-inhibitory effect of p53 (Tasdemir et al., 2008b). However, the exact molecular mechanism by which cytoplasmic p53 inhibits autophagy remains to be elucidated. It seems that cytoplasmic p53 inhibits autophagy through a mechanism different from its function in apoptosis, since BH3-only proteins (such as Beclin-1) are autophagy inducers, but not suppressors (Maiuri et al., 2007). Current research highlights the involvement of the mTOR pathway in regulating cytoplasmic p53-induced autophagy. In HCT116 *p53* ^−/−^ cells with higher basal levels of autophagy, S6K, an mTOR substrate, was hypophosphorylated, whereas AMPK and its substrate ACC were hyperphosphorylated. In addition, cytoplasmic, but not nuclear, p53 inhibited AMPK (reflected by reduced phosphorylation of AMPK, ACC, and TSC2) and activated mTOR (reflected by an increased phosphorylation of S6K) to suppress autophagy (Tasdemir et al., 2008b). These results indicate that cytoplasmic p53 can regulate the AMPK-mTOR axis, but the molecular details remain unclear. Since cytoplasmic p53 can regulate cellular processes by modulating protein-protein interactions, it is important to identify novel cytoplasmic p53-binding proteins, which are involved in controlling mTOR activity, to uncover the exact role of cytoplasmic p53 in regulating the mTOR pathway.

### Regulation of p53 by the mTOR Pathway

The coordination of growth signals and stresses is also adjusted by the reverse regulation of p53 by the mTOR pathway. The role of the mTOR pathway in regulating p53 activity is complex, and is mainly focused on modulating the protein levels of p53 and/or MDM2, a negative regulator of p53. Activating the mTOR signaling by growth factors, such as IGF-1 and hepatocyte growth factor (HGF), induces MDM2 translation in a PI3K-AKT dependent manner. On the other hand, inhibition of mTORC1 by rapamycin downregulating MDM2, induces p53-dependent apoptosis, and sensitizes cancer cells to chemotherapy (Moumen et al., 2007; Du et al., 2013). Additionally, the treatment of Torin1 (an inhibitor of both mTORC1 and mTORC2) or PF-04691502 (a dual PI3K/mTOR inhibitor) increases the expression of p53 protein via inhibition of mTOR signaling (Ekshyyan et al., 2013; Herzog et al., 2013; Garbern et al., 2020). However, in Tsc1 or Tsc2 deletion MEF cells, constitutive mTOR activation promotes the association of p53 mRNA with polysomes to induce its translation (Lee et al., 2007).

**FIGURE 2:**
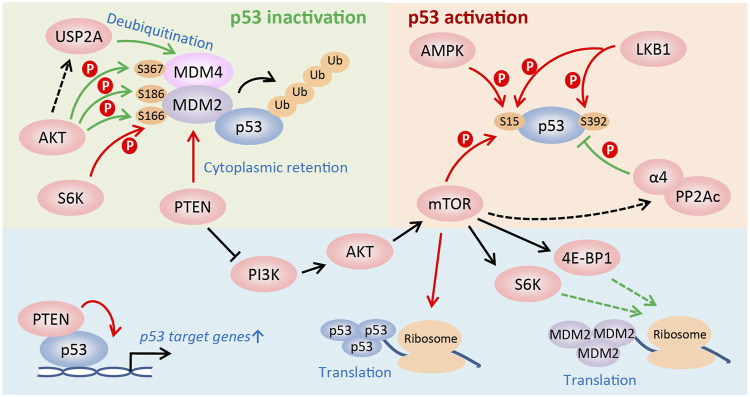
Regulation of p53 activity by the mTOR pathway. The PI3K/AKT/mTOR pathway regulates the activity of p53 at multiple levels, such as translational and post-translational levels, etc. The mTOR pathway is able to either positively or negatively regulate p53 activity in a cell type and stress-dependent manner. See text for details (Red arrow: to promote p53 activity; green arrow: to suppress p53 activity).

In addition to the modulation of p53 activity via changing MDM2 or p53 protein synthesis, the mTOR signaling regulates p53 activity at post-translational levels. First, in PTEN-depleted cells, mTORC1 and mTORC2 compete with MDM2 to bind p53 and phosphorylate it at Ser15, which is the first step to activate p53, leading to PTEN-loss-induced cellular senescence (PICS) (Jung et al., 2019). However, PTEN activates p53 and sensitizes tumor cells to chemotherapy by retaining MDM2 in the cytoplasm (Mayo et al., 2002). And PTEN also promotes p53 transcription activity by regulating its DNA binding independent of MDM2 (Freeman et al., 2003). Second, upon glucose deprivation, phosphorylation of p53 at Ser15 by AMPK leads to cell cycle arrest (Jones et al., 2005). Furthermore, LKB1, the upstream activator of AMPK, directly or indirectly phosphorylates p53 at Ser15 and Ser392, and activates the transcription of *p21* following UV treatment (Zeng and Berger, 2006). Third, AKT promotes p53 degradation by directly phosphorylating MDM2 on Ser166 and/or Ser186, which facilitates the nuclear translocation of MDM2 (Mayo and Donner, 2001; Zhou et al., 2001) and stabilizes it (Feng et al., 2004). Particularly, *in vivo* studies using Mdm2^S183A^ mice recently showed that AKT phosphorylation of Mdm2 at Ser183 (the murine equivalent of human Ser186) suppresses p53-mediated senescence, facilitates ROS-induced tumorigenesis, and has no effects on DNA damage response induced by radiation (Chibaya et al., 2021). Moreover, AKT also regulates phosphorylation of MDM4, which complexes with MDM2 to degrade p53, at Ser367 to stabilize it and consequently inactivate p53 (Lopez-Pajares et al., 2008; Pellegrino et al., 2014). Additionally, AKT may protect MDM4 from proteolysis by inducing the ubiquitin-specific protease 2a (USP2A) to deubiquitinate it (Allende-Vega et al., 2010; Calvisi et al., 2011; Pellegrino et al., 2014). Fourth, in addition to phosphorylating MDM2 on Ser166, S6K1 interacts strongly with MDM2 and inhibits MDM2-mediated p53 degradation in response to DNA damage (Lai et al., 2010). Finally, α4, a nonanalytic subunit of protein phosphatase 2A (PP2A), dephosphorylates p53 and suppresses apoptosis by inhibiting expression of p53 target genes, such as NOXA and p21 (Kong et al., 2004). Rapamycin treatment disrupts the association of α4 with PP2Ac, the catalytic subunit of PP2A, to suppress the phosphatase activity of PP2A (Murata et al., 1997; Inui et al., 1998; Kong et al., 2004), indicating that activating mTOR signaling promotes p53 dephosphorylation and represses its activity. Collectively, the mTOR pathway is able to either positively or negatively regulate p53 activity in a cell type- and stress-dependent manner (Figure 2).

## Conclusions and Future Perspectives

In summary, the coordinated regulation of the tumor suppressor p53 and the mTOR pathway is critical for cells and organisms to maintain homeostasis in response to various stimuli. p53 controls the mTOR pathway at multiple levels: 1) p53 directly regulates several signaling mechanisms in the mTOR pathway; 2) miRNAs, downstream of p53, regulate the mTOR pathway at the post-transcriptional level; 3) cytoplasmic p53 may control the AMPK-mTOR axis to inhibit autophagy by protein-protein interaction. In contrast, the mTOR pathway regulates p53 activity mainly by monitoring the interaction between p53 and its E3 ubiquitin ligase, MDM2. Although some cross talk between p53 and the mTOR pathway has been addressed, many fundamental questions remain unanswered such as: 1) Are the other regulators or components of the mTORC1 complex, the transcriptional targets of p53? 2) How does cytoplasmic p53 activate AMPK and suppress mTOR? 3) What is the precise role of p53 in regulating mTORC2, which has been poorly studied? 4) Does mTOR or its downstream effectors directly phosphorylate other sites of p53 in addition to Ser15 and regulate p53 function under physiological or “stress” conditions? 5) Do the *in vitro* findings truly indicate those *in vivo* physiological and pathological conditions (genetically modified mouse models and clinical patient samples)? The answers to these questions will advance our current understanding of the manner in which the cross talk between p53 and mTOR pathways regulates tumorigenesis.

Interestingly, activating p53 and inhibiting mTOR may be an effective strategy for combating coronaviruses (CoVs) such as COVID-19-causing SARS-CoV-2. During viral infection and replication, mTOR is activated and promotes type-I interferon expression in the presence of MyD88, TLR9, and IRF-7. mTOR inhibitors suppress viral infection and replication in the early stages (Ramaiah, 2020). Recent studies have indicated that several mTOR inhibitors, such as rapamycin and metformin, are potential COVID-19 inhibitors (Gordon et al., 2020; Sharma et al., 2020). However, p53 is an anti-viral factor that is degraded by the E3 ubiquitin ligase RCHY1 upon SARS-CoV infection (Ma-Lauer et al., 2016). Since p53 target genes in the mTOR pathway are mainly negative regulators of mTORC1, p53 activators, such as Nutlin-3a, could help in inhibiting SARS-CoV-2 replication by suppressing mTOR activity.
